# Intrathecal Triamcinolone for Lumbar Degenerative Disease: A Single-Center Retrospective Cohort of 499 Patients

**DOI:** 10.3390/jcm14197057

**Published:** 2025-10-06

**Authors:** Stefan Aspalter, Nico Stroh-Holly, Johanna Burgholzer, Wolfgang Senker, Milan Vosko, Philip Rauch, Andreas Gruber, Harald Stefanits

**Affiliations:** Department of Neurosurgery, Kepler University Hospital, Johannes Kepler University Linz, 4020 Linz, Austria

**Keywords:** lumbar spinal stenosis, intrathecal triamcinolone, degenerative spine disease, corticosteroid therapy, pain management

## Abstract

**Background/Objectives**: While epidural and periradicular corticosteroid injections are well-established treatments for degenerative spinal conditions, intrathecal administration of glucocorticoids remains uncommon and under-researched. To our knowledge, this is the first large contemporary dataset on intrathecal triamcinolone in degenerative lumbar disorders. This study retrospectively analyzes clinical outcomes and complication rates associated with this treatment. **Methods**: We reviewed patients who received intrathecal injections of triamcinolone for lumbar degenerative spinal diseases between May 2023 and June 2024. Data were extracted from electronic records and included demographics, indication, application method (freehand or CT-guided), dosage, symptom relief, and complications. **Results**: A total of 722 intrathecal injections were performed (499 patients). The most common indication was lumbar spinal canal stenosis (94.0%). Punctures were performed freehand in 68.4% of the injections; 80 mg of triamcinolone was administered in 71.2%. Follow-up data were available for 528 injections. After 87.3% of these, symptom improvement (binary yes/no) after injection was reported. Duration of benefit was documented after 144 injections: 39.6% reported a relief lasting up to six months, and 25% up to one month. Four complications (0.6%) occurred: one post-puncture headache, one pain aggravation, one case of shortness of breath, and one intracranial subdural hygroma. All were managed conservatively. **Conclusions**: Despite limited data quality, including missing/non-standardized follow-up and the lack of standardized pain scales for follow-up, this large retrospective cohort provides preliminary evidence that intrathecal triamcinolone may be a safe and effective treatment option for lumbar degenerative spinal disorders, with pain relief observed in the majority of cases. Given the inherent limitations of retrospective Level IV evidence, prospective controlled studies are warranted to further evaluate its role compared to other interventional pain therapies.

## 1. Introduction

Epidural and periradicular application of corticosteroids is widely accepted as a treatment option in degenerative spine diseases like disk herniation or spinal stenosis and has been the subject of extensive research [1,2,3,4,5].

In contrast, the intrathecal administration of glucocorticoids for pain management in degenerative spinal diseases remains largely unexplored to date and is used relatively infrequently. Only limited data exist on the use of intrathecal corticosteroids for patients with degenerative spine disease and sciatica, and most of these studies date back to the 1960s and 1970s [6,7,8].The transition of intrathecal to epidural injection of corticosteroids began in 1972, as Winnie et al. reported that epidural methylprednisolone acetate led to significant pain relief in patients with sciatica [7,9]. However, these historical studies are of limited generalizability to current practice, as they were conducted in small and heterogeneous patient cohorts, often without standardized pain assessment tools, and prior to the establishment of today’s interventional pain management standards and safety protocols.

Recent systematic reviews and guidelines suggest that epidural steroid injections are supported by substantial evidence for short-term pain relief, particularly when image-guidance is used (interlaminar, transforaminal, caudal approaches) [10]. However, risks such as inadvertent intravascular injection, infection, neurological injury, or even rare serious events (e.g., meningitis, stroke) are documented [11]. For intrathecal administration, by contrast, much less recent and robust data exist regarding safety, technique standardization, and long-term outcomes. Thus, comparing intrathecal approaches to well-characterized epidural counterparts is essential to properly assess the risk-benefit profile. Considering pain and intrathecal glucocorticoid injection, more recent studies only investigated the usage in patients with postherpetic neuralgia or complex regional pain syndrome, and most of these had relatively small study populations [12,13,14,15].

At our institution, intrathecal glucocorticoids (e.g., triamcinolone) have been routinely used for decades in the treatment of lumbar degenerative disorders. Given this ongoing practice, a critical evaluation of this therapeutic approach is warranted, particularly regarding potential complications. Therefore, the primary objective of this study is to retrospectively assess clinical outcomes following intrathecal injections, with the primary outcome being patient-reported symptom improvement. Secondary outcomes include the incidence and type of complications observed.

## 2. Materials and Methods

The retrospective single-center study was conducted at the Department of Neurosurgery, Kepler University Hospital, Linz, Austria, and the ethical approval was obtained from the local Ethics Committee of the Federal State of Upper Austria (EK-No.: 1297/2024).

**Figure 1:**
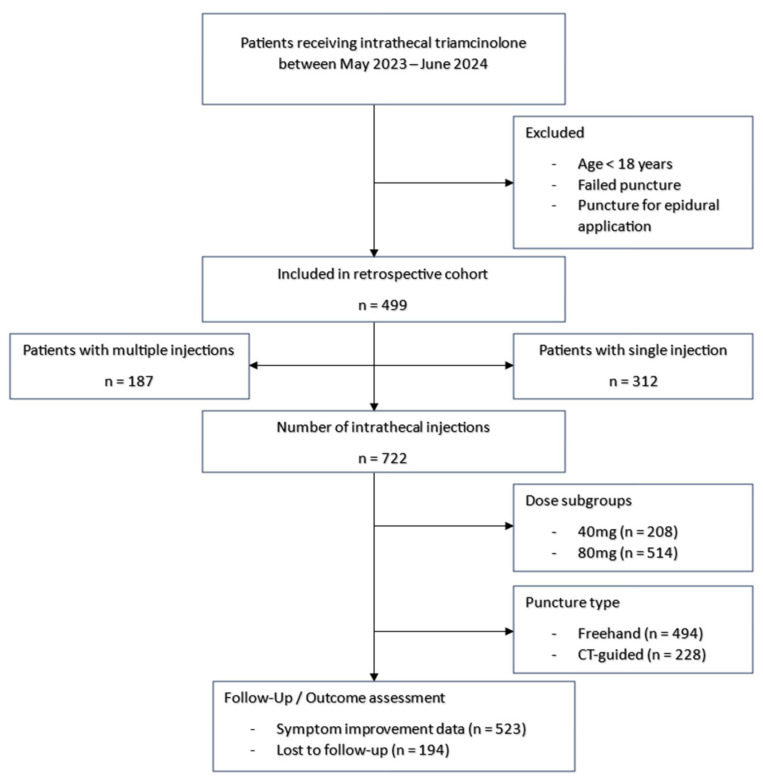
STROBE-style flow diagram, describing the retrospective cohort of patients receiving intrathecal triamcinolone between May 2023 and June 2024 with inclusion and exclusion criteria.

Study design: This retrospective study included patients who received the intrathecal administration of triamcinolone acetonide for the treatment of lumbar degenerative spinal diseases at our department between May 2023 and June 2024. Inclusion criteria were an intrathecal administration of triamcinolone acetonide (Volon A; Dermapharm GmbH, Vienna, Austria) for lumbar degenerative spinal disease via lumbar puncture between May 2023 and June 2024. Exclusion criteria were age under 18 years, failed puncture, and punctures for epidural application of triamcinolone acetonide. A STROBE flow diagram was created to illustrate the selection process of the study population, including inclusion and exclusion criteria (see Figure 1).

Procedure: The indication for intrathecal administration of triamcinolone was determined during an outpatient evaluation. The injection itself was performed during a subsequent inpatient admission. Informed consent was obtained. The puncture was performed either freehand or under computed tomography (CT) guidance, depending on anatomical conditions. CT-guided procedures were performed in cases where a prior freehand attempt was unsuccessful or in patients with anatomical variations or previous spinal surgeries involving implanted instrumentation that made freehand puncture challenging. Freehand punctures were performed when standard lumbar anatomy allowed safe access.

According to internal clinical practice, 40 mg was used in patients with diabetes mellitus or other disorders of glucose metabolism, while 80 mg was used in patients without such pre-existing conditions. Lumbar punctures were performed using a 22 G traumatic spinal needle at the intended lumbar interspace levels L4/5 or L5/S1. No adjuncts such as local anesthetics or saline were co-administered with triamcinolone. Following the lumbar puncture, patients were required to remain on bed rest for six hours and were discharged either on the same day or the following day. In general, no fixed follow-up appointments were scheduled. Patients were advised to return to the outpatient clinic only in case of persistent or recurrent symptoms.

Data collection: Assessed parameters were patient demographics, indication for intrathecal application of triamcinolone, number of applications (in case of patients receiving more than one injection within the study period), type of application (freehand / CT-guided), dose (40 mg or 80 mg), subjective clinically significant pain relief after injection (yes/no). Pain relief was assessed by patient self-report during the inpatient stay immediately following the injection, or by a neurosurgeon during a follow-up appointment. No standardized pain scales, such as VAS or NRS, were used; therefore, outcomes are descriptive. Duration of pain improvement and occurrence of complications were also recorded.

## 3. Results

A total of 499 patients were included in the study. Patient characteristics are shown in Table 1. In cases where patients received intrathecal therapy with triamcinolone multiple times, these administrations were also included. In total, 722 administrations of triamcinolone were considered in the study, as some patients received more than one injection during the study period. Mean patient age was 71.0 years old at the time of the first treatment. 49.5% (247) patients were female and 50.5% (252) were male. The punctures were performed for lumbar spinal canal stenosis (94.0%), neuroforaminal stenosis (1.6%), adjacent segment degeneration (1.0%), degenerative spondylolisthesis (2.2%), synovial cysts (0.4%), and disk prolapse (0.8%).

The intrathecal application of the drug was carried out via freehand puncture in 68.4%, or CT-guided puncture in 31.6%. Depending on pre-existing conditions, either 40 mg (28.8%) or 80 mg (71.2%) of triamcinolone was administered.

Follow-up data were available after 391 (54.2%) of all 722 injections. In 345 of 391 (88.2%) cases of intrathecal triamcinolone administration, the patients reported a subjective clinically significant improvement in pain symptoms following treatment. In 46 of 391 cases (11.8%), the intervention did not lead to any pain relief. For the remaining 331 punctures, the effectiveness of the administered medication could no longer be assessed retrospectively due to a lack of follow-up or documentation.

Of the patients who reported a significant clinical improvement, documentation regarding the duration of treatment benefit was available after 144 punctures (19.9% of 722 total punctures). In 57 of 144 (39.6%) cases, symptom relief lasted up to 6 months. In 36 of 144 (25.0%) cases, patients experienced relief for up to one month. Exact details of the symptom improvement are shown in Table 2.

**Figure 2:**
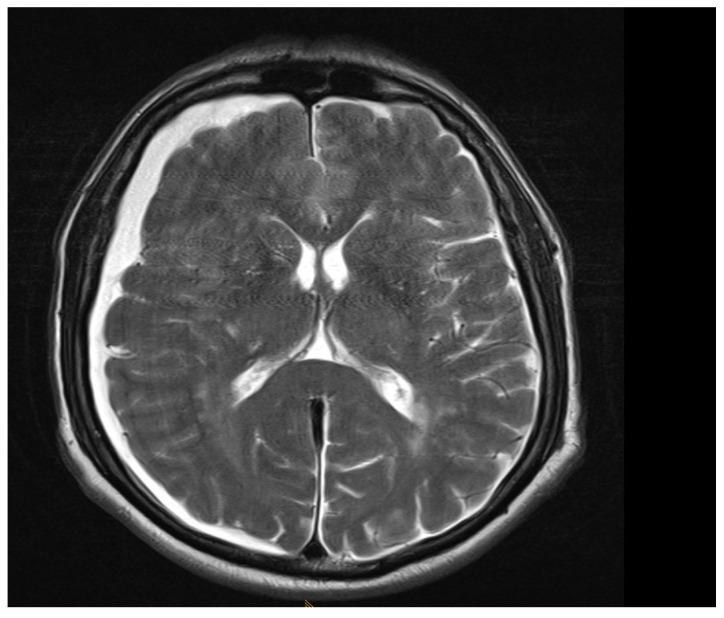
Axial T2 MRI, showing subdural hygroma on the right side. The image was acquired 1 month after intrathecal injection due to persistent headache.

**Figure 3:**
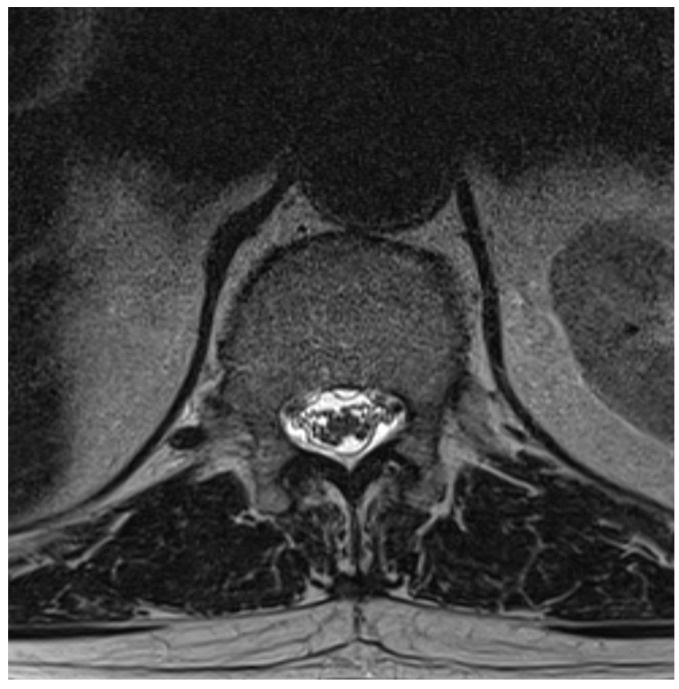
Axial T2-weighted MRI at L1, showing epidural cerebrospinal fluid accumulation both ventral and dorsal to the thecal sac.

**Figure 4:**
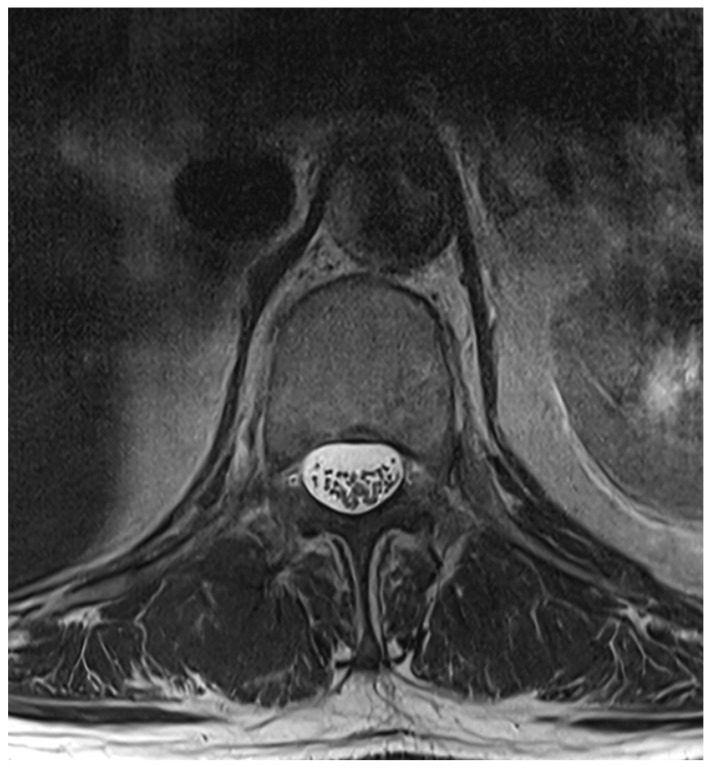
Axial T2 weighted MRI at L1, 6 weeks after blood patch. No epidural cerebrospinal fluid collections can be distinguished anymore.

Among the 722 punctures, complications occurred in four cases (0.6%), see Table 3. Three of these complications arose after the first administration and one after the second. Reported complications included one case of post-puncture aggravation of pain, and one case of post-puncture headache. Both cases were managed conservatively using analgesics. In one case, shortness of breath occurred. In this patient, a cardiopulmonary event was ruled out, which required performing blood sampling as well as a CT-angiography of the pulmonary arteries. In another case, an intracranial subdural hygroma occurred, see Figure 2. No neurological deficits occurred, but due to persistent headache, an MRI scan was performed which revealed the diagnosis. The patient was readmitted for inpatient treatment for another 5 days 1 month after initial puncture. An MRI scan of the lumbar scan revealed epidural accumulations of cerebrospinal fluid at the thoracolumbar junction (Figure 3). Bed rest was ordered for 3 days. 2 days after readmission, a CT-guided lumbar blood patch was performed at the level L2 with 35 mL of blood, and oral analgesics were administered. The patient was discharged after five days. At subsequent follow-up visits, the patient reported no further headaches, and an MRI performed six weeks later demonstrated the complete resolution of the epidural cerebrospinal fluid collections (Figure 4).

Table 4 presents a comparative analysis of improvement rates stratified by dosage, puncture type, and indication, restricted to each patient’s first injection. Statistical testing was performed using Pearson’s Chi-squared test. No significant differences were observed between groups for dosage, puncture type, or indication.

## 4. Discussion

While epidural and periradicular corticosteroid injections are widely used and well-studied therapeutic options for spinal pain syndromes, intrathecal glucocorticoid application remains relatively underexplored. This study, comprising 499 patients and 722 injections, represents the only contemporary study of this topic and provides to our knowledge the largest dataset on this specific intervention.

Our findings indicate that intrathecal triamcinolone is a generally safe and effective therapeutic approach in the treatment of pain related to degenerative lumbar spine disease. A substantial proportion of patients (87.3% of those with follow-up data) reported symptomatic improvement following the intervention. These results align with historical data from smaller studies, most of which date back several decades, supporting the analgesic potential of intrathecal corticosteroids in patients with radicular or spinal-origin pain [6,7,8]. In our department, intrathecal triamcinolone has long served as the primary interventional treatment for symptomatic central lumbar spinal canal stenosis, which was also the predominant indication in our cohort. While these data do not constitute high-level evidence, they suggest that the therapy is both feasible and clinically valuable in everyday practice. As the use of triamcinolone in this context is off-label, prospective studies—particularly randomized trials—would require substantial regulatory approval and funding, which has thus far impeded their realization. Nonetheless, our findings provide important hypothesis-generating insights and underscore the need for controlled trials to define more precisely the role of intrathecal triamcinolone in comparison with established conservative and interventional strategies.

However, one of the main limitations of our study is an inconsistent follow up and also the lack of standardized pain rating scales like the Visual Analogue Scale or Numeric Rating Scale. Both limitations are since the typical patient course is that after indication of the procedure and subsequent injection, patients are not routinely followed up. The fact that patients who still suffer from symptoms after injections are probably more likely to show up at the department again, would indicate that probably the patients with missing follow-up are patients who are sufficiently pain relieved. However, other factors such as long distance, lack of motivation to attend follow-up, dissatisfaction with treatment, ongoing care at other facilities, socioeconomic issues like financial constraints or work commitments, as well as organizational challenges including missed appointments or communication problems, may also have contributed to the loss to follow-up, potentially biasing our results.

Interestingly, most injections were performed via a freehand technique (68.4%), suggesting that intrathecal administration is feasible in most patients even in the presence of degenerative spinal disorders, without requiring image guidance. CT-guided punctures were primarily reserved for patients with anatomical challenges, such as prior spinal instrumentation or difficult anatomy. This flexibility in approach supports the adaptability of the technique in routine clinical practice.

Despite its efficacy, the duration of symptom relief following intrathecal administration varied. Among cases with documented follow-up on pain relief duration, approximately 40% reported sustained benefit for up to six months, while 25% experienced only transient improvement lasting up to one month. These figures highlight the variability in treatment response and suggest that while intrathecal triamcinolone may offer significant temporary relief for many patients, it may not be a long-term solution for all.

The safety profile of intrathecal triamcinolone observed in our cohort, demonstrating a low complication rate of 0.6% (all managed conservatively), appears favorable when compared to recent data on epidural and periradicular steroid injections. Several recently published studies report complication rates ranging between 2.4% and 9.6%, predominantly minor adverse events such as postural headaches (0.5–1%), injection site pain, and transient neurological symptoms. More serious but rare complications include epidural hematoma (0.01–0.02%), infections (e.g., epidural abscess), and nerve root injury [16,17,18]. While the risk of catastrophic events remains low, these complications underscore the need for caution in patient selection and procedural expertise. Our lower observed complication rate lends support to the relative safety of intrathecal administration in selected patients, though prospective controlled data are necessary for definitive conclusions.

A potential difference contributing to the efficacy of intrathecal triamcinolone compared to epidural or periradicular injections is the more direct distribution of the corticosteroid around compressed nerve roots within the cerebrospinal fluid. This proximity may enhance the drug’s anti-inflammatory effects at the site of pathology. However, the clinical relevance and consequences of this differential distribution have not yet been investigated. The therapeutic benefit observed in our cohort likely reflects the well-established anti-inflammatory actions of corticosteroids, which reduce local edema and neural irritation, contributing to symptom relief. Further mechanistic studies are needed to clarify these effects in the context of intrathecal administration.

Comparing the complications to other studies which investigated risks of lumbar puncture, a 2018 meta-analysis by Nath et al. reported a frequency of 11% for post-puncture headaches and a 3.0% need for a blood patch when conventional needles were used for lumbar puncture [19]. The occurrence of a subdural hygroma however is a way more less frequent complication than just post-puncture headaches, and must be considered a serious side effect. Subdural hygromas following lumbar puncture are very rare complications, with an estimated incidence below 0.01% in most patient populations undergoing this procedure [20,21].

It must be noted that due to the retrospective design, patients who may have developed slight post-puncture headaches after discharge might not have returned to our department, leading to potential underreporting. Due to the fact that our hospital is the major center for neurological and neurosurgical diseases in the federal state, however, it can be assumed that the majority of patients who would have developed severe symptoms or complications after injection, would have been seen at our department again as well. Compared to the known complication rates of other neuraxial techniques such as lumbar puncture or spinal anesthesia, these figures suggest a favorable safety profile, although the risk of rare but serious complications like hygroma emphasizes the need for careful patient selection and informed consent.

Our results also compare favorably to those of Russegger et al. [22] who reported a 13% rate of transient minor complications following intrathecal triamcinolone administration in post-discectomy patients. In contrast, our study showed a markedly lower complication rate and a substantially higher rate of symptomatic improvement, suggesting that intrathecal corticosteroid therapy may be more effective and safer than previously assumed.

In our cohort, most patients were treated for lumbar spinal canal stenosis (94%), with only a minority presenting with other degenerative disorders such as synovial cysts or disk herniation. As a result, the applicability of our findings is greatest for lumbar spinal stenosis, though they may also hold relevance for other degenerative conditions.

While our results demonstrate a favorable safety profile and promising symptom improvement with intrathecal triamcinolone, direct comparisons with epidural and periradicular steroid injections should be interpreted with caution. The absence of a control group and the lack of standardized outcome measures in our study prevent definitive conclusions about relative efficacy and safety. Thus, although complication rates in our cohort were low, our findings remain preliminary. Further prospective controlled trials are needed to rigorously assess how intrathecal triamcinolone compares to established modalities.

## 5. Limitations

Several major limitations are encountered. The main factor is the retrospective design, which inherently carries the risk of incomplete data. Follow-up data were missing or incomplete for a notable proportion of cases (approximately 27% lacked data on treatment effect, and nearly 80% lacked documentation on duration of benefit), limiting the ability to fully assess long-term outcomes. Due to lack of data, systematic subgroup analyses to define differential treatment effects (e.g., by comorbidities, anatomical severity, or previous interventions) were not possible. Additionally, the lack of a control group and standardized follow-up prevents a direct comparison to alternative treatment modalities such as epidural steroid injections or conservative management. Furthermore, the lack of standardized pain assessment tools (e.g., VAS, NRS) limits comparability with other published outcomes.

Nevertheless, this study provides valuable insights into a long-standing, yet underreported, clinical practice. Given the widespread use of intrathecal corticosteroids at our institution and the overall favorable outcome profile observed, further prospective studies are warranted. Such studies should aim to standardize follow-up, assess functional outcomes, and compare intrathecal therapy directly with other pain management strategies. This would help delineate its exact role within the spectrum of interventional pain treatments for degenerative spinal disease.

## 6. Conclusions

Although this retrospective analysis has clear limitations—such as incomplete and non-standardized follow-up, the absence of validated pain scores, and the lack of a control group—it nevertheless represents a large single-center cohort (499 patients; 722 intrathecal triamcinolone injections) and offers valuable exploratory insights. Our observations indicate that intrathecal triamcinolone may contribute to clinically relevant symptom relief in the majority of patients, with a low rate of short-term complications (0.6%, all managed conservatively). In light of the scarcity of contemporary data on this subject, these findings should be considered preliminary and hypothesis-generating. Rigorous prospective studies with standardized outcome measures are needed to substantiate these results and to more clearly delineate the therapeutic role of intrathecal triamcinolone in degenerative lumbar spinal disease.

## Figures and Tables

**Table 1:** Descriptive characteristics of all patients, including a comparison of baseline characteristics between patients with and without follow-up.

	*n*	%	Follow-Up	No Follow-Up
Total patients	499	100		
Gender				
Female	247	49.5	154 (52.0)	93 (46.0)
Male	252	50.5	144 (48.0)	108 (54.0)
Age in years, median [IQR]	72	[66, 78]	72 [66, 79]	72 [66, 78]
Triamcinolone Injections per patient during study period				
Total	722	100	391	331
1	347	70.0	190 (64.0)	157 (78.0)
2	113	23.0	75 (25.0)	38 (19.0)
3	23	4.6	18 (6.0)	5 (2.5)
4	7	1.4	6 (2.0)	1 (0.5)
5	3	0.6	3 (1.0)	0 (0.0)
6	5	1.0	5 (1.7)	0 (0.0)
7	1	0.2	1 (0.3)	0 (0.0)
Indication for Injection				
Spinal canal stenosis	396	79.0	311 (80.0)	262 (79.0)
Others	103	21.0	71 (18.0)	64 (19.0)
Dosage				
40 mg	208	29.0	110 (28.0)	98 (30.0)
80 mg	514	71.0	281 (72.0)	233 (70.0)
Puncture type				
Freehand	494	68.0	269 (69.0)	221 (67.0)
CT-guided	228	32.0	122 (31.0)	106 (33.0)

**Table 2:** Follow-up data for symptom improvement (per injection).

	*n*	%
Available follow-up data (any improvement)	391	100
Symptom improvement after injection		
Yes	345	88.2
No	46	11.8
Available Follow-up data (duration of improvement)	144	100
Duration of symptom improvement		
Up to 1 day	6	4.2
Up to 1 week	23	16.0
Up to 1 month	36	25.0
Up to 6 months	57	39.6
Up to 1 year	15	10.4
More than 1 year	7	4.9

**Table 3:** Characteristics of the complications occurred.

	Complication	Time of Occurrence After Injection	Treatment	Outcome at Last Contact
Patient 1	Post-puncture aggravation of pain	Within 1 day (inpatient)	Oral Analgesics, after ruling out of epidural hematoma via MRI	Pain-free at 3-month FU
Patient 2	Post-puncture headache	Within 1 day (inpatient)	Oral Analgesics	No headache at 1-month FU
Patient 3	Shortness of breath	Within 2 h (inpatient)	Self-remitting; ruling out of cardiopulmonary event by CT-angiography and blood sampling	No complaints at dismission
Patient 4	Intracranial subdural hygroma (persistent headache, no neurological deficits)	Within 1 month (inpatient)	Lumbar CT-guided blood patch, oral analgesics	No headache at 6 week FU

CT = computed tomography, MRI = magnetic resonance imaging, FU = follow up.

**Table 4:** Comparative analysis of improvement rates stratified by dosage, puncture type, and indication, restricted to each patient’s first injection. Absolute numbers are given with percentages in parentheses. Statistical testing was performed using Pearson’s Chi-squared test. Analyses were based on n = 298 patients; 201 patients had missing values for the variable improvement.

		Improvement	No Improvement	*p*-Value
**Dosage**	40 mg	30 (88.0)	4 (12.0)	**>0.9**
	80 mg	184 (90.0)	21 (10.0)	
**Puncture type**	Freehand	194 (89.0)	24 (11.0)	**>0.9**
	CT-guided	71 (89.0)	9 (11.0)	
**Indication for Injection**	Spinal canal stenosis	210 (88.0)	29 (12.0)	**0.3**
	Others	55 (93.0)	4 (6.8)	

## Data Availability

The data that support the findings of this study are available from the corresponding author, upon reasonable request.

## Abbreviations

The following abbreviations are used in this manuscript:

CSF	Cerebrospinal Fluid
CT	Computed tomography
MRI	Magnetic Resonance Imaging
NRS	Numeric Rating Scale
VAS	Visual Analogue Scale
